# Sensitivity and Selectivity of Two Commercially Available Media for *Legionella* spp. Recovery from Environmental Water Samples

**DOI:** 10.3390/pathogens9070523

**Published:** 2020-06-29

**Authors:** Savina Ditommaso, Monica Giacomuzzi, Gabriele Memoli, Jacopo Garlasco, Carla M. Zotti

**Affiliations:** Department of Public Health and Pediatrics, University of Turin, 10126 Turin, Italy; monica.giacomuzzi@unito.it (M.G.); gabriele.memoli@unito.it (G.M.); jacopo.garlasco@unito.it (J.G.); carla.zotti@unito.it (C.M.Z.)

**Keywords:** *Legionella* spp., culture media, environmental monitoring, quality control

## Abstract

The quality control of culture media used for *Legionella* spp. isolation and enumeration is paramount to achieve a satisfactory degree of comparability among water testing results from different laboratories. Here, we report on a comparative assessment of the sensitivity and selectivity of MWY and BCYEα media supplied by two different manufacturers (i.e., Xebios Diagnostics GmbH and Oxoid Ltd) for the detection of *Legionella* spp. from environmental water samples. Even though our analysis showed an excellent agreement between the recovery rates of the four media tested (90.5%), the quantitative recovery of *Legionella* spp. colonies using Xebios media was significantly greater than that achieved by Oxoid media (*P* = 0.0054). Furthermore, the sensitivity of detection was significantly higher when samples were plated on MWY ^Xebios^ agar (*P* = 0.0442), while the selectivity of MWY appeared to be the same regardless of the manufacturer. Furthermore, MWY^Xebios^ agar favored the growth of much larger colonies compared to those observed on MWY^Oxoid^ agar. Finally, MWY^Xebios^ medium enhanced the recovery of non-*pneumophila Legionella* species. Collectively, our findings demonstrate that quality control is crucial to ensure high selectivity and sensitivity of the culture media used for the detection and enumeration of *Legionella* spp. from environmental water resources. As water remediation measures strictly depend on *Legionella* spp. recovery, culture protocol standardization, as well as quality control of the culture media, is essential to achieve intra- and interlaboratory reproducibility and accuracy.

## 1. Introduction

The most common protocol for environmental surveillance of *Legionella* spp. involves the use of buffered charcoal yeast extract agar enriched with 1 g/L alpha-ketoglutarate agar (BCYEα). Although this method has a proven record of effectively isolating and enumerating *Legionella* species from environmental or clinical specimens, its sensitivity and selectivity are often hampered by the presence of contaminating flora in the water samples, which may influence the final count of *Legionella* spp. due to overgrowth or inhibition. Therefore, culture on selective agar media—i.e., BCYEα supplemented with glycine, vancomycin, polymyxin B, cycloheximide (GVPC) or with glycine, polymyxin B, vancomycin, anisomycin, bromothymol blue, and bromocresol purple (MWY)—that is capable of inhibiting most non-*Legionellaceae* bacteria is the preferred solution to isolate *Legionella* spp. from environmental specimens [1,2,3]. 

Given that the recovery of *Legionella* spp. strictly depends on the type of agar being used, quality-assured culture media for water testing are key to consumer safety. However, the overall performance of commercially available nonselective (BCYEα) and selective (MWY and GVPC) media for *Legionella* spp. isolation has only been partially addressed. In this regard, the last studies on the quality assurance of these media date back to 2004 [4] and 2010 [5]. 

According to the literature, these types of media are very difficult to prepare, store, and test as minor differences in pH, cation content, and agar composition can heavily influence growth rates, plating efficiency, and colony formation [6,7,8]. Thus, the quality control of culture media used for *Legionella* spp. detection is now, more than ever, essential to achieve a satisfactory degree of comparability among water-testing results from different laboratories.

For over two decades, our group has been conducting *Legionella* spp. testing in numerous hospitals and health facilities in the Piedmont region of Italy. Furthermore, since 2007, our laboratory has been a permanent member of the External Quality Assessment (EQA) scheme for *Legionella* spp. isolation from water, which relies on the use of the BCYEα and MWY media manufactured by Oxoid Ltd. (Basingstoke, UK), with satisfactory z-scores of performance throughout. In particular, by conducting parallel seeding experiments, we have previously found that BCYEα allows a higher yield and recovery rate of *Legionella* spp. positive samples (93%) compared to that obtained with MWY (78%). Based on these findings, we were the first in 2011 [9] to recommend the use of BCYEα as a nonselective medium, in addition to MWY or GVPC, for optimal detection of *Legionella* spp. in environmental water samples. The combined use of one selective and one nonselective media for improved *Legionella* spp. detection was later on incorporated in the second edition of ISO 11731 [10].

Besides the recovery rate, we have also been actively involved in evaluating the sensitivity and selectivity of nonselective vs. selective media for the isolation and enumeration of *Legionella* spp., an investigation that has been more recently extended to BCYEα-AB media (Ditommaso et al., unpublished data). 

In this study, we report on a comparative assessment between the sensitivity and selectivity of the aforementioned media from Oxoid Ltd. (Basingstoke, UK) and the corresponding media manufactured by Xebios Diagnostics GmbH (Düsseldorf, Germany) for the detection of *Legionella* spp. from environmental water samples. 

## 2. Results 

### 2.1. Overall Results

A total of 148 water samples were cultured on MWY and BCYEα agar produced by two different manufacturers (i.e., Xebios and Oxoid). Bacteria were isolated from 70/148 samples (47.3%) using either type of medium, showing comparable levels of *Legionella* spp. detection. Specifically, we detected 64 (43.2%) and 62 (41.9%) positive cultures using Xebios and Oxoid mediums, respectively, with excellent agreement between the two brands (90.5%; κ = 0.807).

According to the Wilcoxon test analysis performed on 148 results obtained with each medium, the detection sensitivity increased when the samples were plated on Xebios medium (*P* = 0.0054, median difference between log-counts: Δ = 0.192, CI_95%_: 0.055, 0.394). Consistently, the 56 concordant positive samples also displayed the highest counts on Xebios agar plates (*P* = 0.0006, Δ = 0.159, CI_95%_: 0.068, 0.295).

### 2.2. Results Relative to Medium Type

To further investigate the differences in the recovery of *Legionella* spp., we assessed the sensitivity and selectivity of Oxoid vs. Xebios alpha-ketoglutarate agar (BCYEα) and bromocresol purple (MWY) in detecting *Legionella* spp. from 148 environmental water samples (Table 1). 

The detection sensitivity was significantly higher in samples plated on MWY^Xebios^ agar compared to that of samples grown on MWY^Oxoid^ agar (McNemar test: *P* = 0.0042). No difference was found between BCYEα^Oxoid^ and BCYEα^Xebios^ media (McNemar test: *P* = 0.03588); no difference in suppressing non-*Legionella* bacteria (i.e., selectivity) was found between the two brands of MWY agar. 

A Wilcoxon signed-rank test comparison (Table 2) revealed a significant difference in the number of *Legionella* spp. colonies, not only between BCYEα^Xebios^ and MWY^Xebios^ media but also between MWY^Oxoid^ and MWY^Xebios^ media. In either case, the recovery of *Legionella* spp. was significantly higher when the samples were plated on MWY^Xebios^ agar.

All data were disaggregated according to medium and manufacturer. Table 3 classifies the samples according to the presence or absence of *Legionella* spp. in MWY and BCYE media from Oxoid (Table 3a) or Xebios (Table 3b). There is a greater agreement between the two Xebios media (47/49) in comparison with that between the two Oxoid media (34/44). 

Pearson’s chi-squared test comparison between Oxoid and Xebios results, after a Deming–Stephan adjustment, revealed a statistically significant difference between the two brands (χ^2^ = 9.0824, *P* = 0.0026; Table 3c). 

The number of *Legionella* spp. colonies (CFU/L) growing or not on BCYEα and MWY is shown in Table 4 and Table 5.

The observation that some water samples were *Legionella* spp. positive on BCYEα medium but negative on MWY agar (7/10 cultures, samples 4–10, Table 4) indicates that the use of selective medium can affect the recovery of non-*Legionella pneumophila* species. Cell concentrations from these water samples ranged from 2.0 × 10^2^ to 3.4 × 10^3^ CFU/L. These results only refer to samples cultured on Oxoid media (Table 4).

Table 5 shows that high levels of background flora can challenge the results: on BCYEα agar, the results were affected by the presence of concomitant background flora that prevented the growth or masked the observation of *Legionella* colonies in BCYEα agar from both manufacturers. No qualitative data are available for these aquatic bacteria. Contaminating non-*Legionella* bacteria were rarely recovered on MWY agar from either manufacturer. 

The influence of the media on the detection time was also assessed. Typically, antibiotics added to the selective media suppress the accompanying flora but at the same time slow down the growth of the target organism. The average colony size of *Legionella* spp. cultured on Xebios media was greater than that of *Legionella* spp. plated on Oxoid medium, especially for bacteria plated onto MWY agar. With regard to colony count, the number of *Legionella* spp. was higher on MWY^Xebios^ agar compared to MWY^Oxoid^. As for the Oxoid media, even though the supplemented antibiotics suppressed the accompanying flora, it slowed down excessively the growth of the target organisms. No particular differences in colony size or count were observed between the two brands of BCYEα agar, as already shown in Table 2 (see also the supplementary materials).

## 3. Discussion 

Several factors may hinder exact *Legionella* spp. quantification in environmental samples: (i) differences in the polycarbonate membrane characteristics (e.g., pore size, batches, fragility, crinkling and electrostatic interactions), (ii) different washing procedures to remove trapped bacteria from the membrane (e.g., shaker/vortex, ultrasound, finger and thumb scraping, or heat or acid treatment), which favor the detection of the microorganism but at the same time may reduce its concentration, and (iii) the choice of the culture medium [11,12,13,14]. 

As for the latter, the parameters affecting its quality are the following: (1) type and quantity of nutrients, (2) redox potential (Eh)—both after preparation and during incubation, (3) initial pH and buffering capacity, (4) water activity, and (5) type and activity of the antimicrobial agents—these can either be supplemented, already be present in the medium components, or accidentally form due to preparation errors, such as excessive heating [6,15]. Further evidence has highlighted several other deficiencies of those selective media that rely on a delicate balance between productive and selective mechanisms [4,8,16,17].

The quality of culture media has a dramatic effect on *Legionella* spp. recovery and counts. To evaluate the contribution of culture media, we checked relative recoveries of *Legionella* spp. from 148 environmental water samples because the response obtained from media plated with collection strains (i.e., quality control protocol) may vary when wild bacterial strains are present. It is known, for example, that virulent *L. pneumophila* are especially salt-sensitive, and that spontaneous mutations in stock strains may result in salt resistance [18]. Consequently, testing culture media using stock strains of bacteria may not always be a valid approach [5]. 

Although several different medium formulations are routinely used to detect *Legionella* spp. from environmental samples [10], there is paucity of studies assessing and comparing their abilities in growing *Legionella* spp. [4,5,9]. Since the quality of the culture medium strongly influences *Legionella* spp. detection and enumeration due to the presence of contaminating flora, here, we have assessed the recovery rates of *Legionella* spp. from hospital water samples using two different brands (i.e., Xebios and Oxoid) of either nonselective BCYEα or selective MWY medium.

Our analysis shows an excellent agreement between the recovery rates of the media from both companies (90.5%). Nonetheless, the quantitative recovery of *Legionella* colonies using Xebios media is significantly greater than that achieved by Oxoid media. Furthermore, the sensitivity of detection is significantly higher when samples are plated on MWY ^Xebios^ agar, while the selectivity of MWY appears to be the same regardless of the manufacturer. Moreover, there is a greater agreement between the two Xebios media compared to that between the two Oxoid media. Additionally, differences in colony size were apparent for the different agars (see supplementary materials). Specifically, the MWY^Xebios^ agar favored the growth of much larger colonies compared to MWY^Oxoid^, and enhanced the recovery of non-*Legionella pneumophila* species. 

As we used four different batches over a one-year period of study, it is highly unlikely that batch-to-batch variability may have played a role in the performance differences that we observed. We hypothesize that other factors such as the presence in the medium of toxic compounds (e.g., metals), growth-promoting factors, or high gel strengths may have influenced the growth and colony size on different types of medium, as previously shown for *L. pneumophila* on BCYEα agar [19].

Collectively, these results highlight significant differences between the performances of media from different manufacturers. Despite generating the same number of positive cultures, the Xebios media generally yielded greater numbers of *Legionella* spp. and larger colony sizes, allowing easier detection. Thus, the use of Xebios culture media is indicated to achieve the highest sensitivity and selectivity when detecting environmentally sampled *L. pneumophila*.

## 4. Methods 

### 4.1. Water Samples

Media were tested using environmental samples obtained from hospital building waterlines. One liter of sample was collected from each site in sterile one-liter plastic bottles. A sodium thiosulphate (100 mg/L) solution was added to the samples to neutralize free chlorine in treated water supplies.

### 4.2. Culture Media

To distinguish between *Legionella* spp. and non-*Legionella* bacteria, two different media were used: 0.1% BCYEα agar and BCYEα agar supplemented with 3 g/L glycine, 50,000 IU of polymyxin B, 0.001 g/L of vancomycin, 0.08 g/L of anisomycin, 0.01 g/L of bromothymol blue, and 0.01 g/L of bromocresol purple (MWY). The formulations of both media conform to ISO 11731 [10]. 

Commercially available agar plates were purchased by Oxoid Ltd. (Basingstoke, UK) or Xebios Diagnostics GmbH (Düsseldorf, Germany). For each batch supplied, each manufacturer provided us with detailed quality control information (e.g., type of bacteria, pH, colony morphology, selectivity, recovery and expected results). The microbiological performance test was carried out in accordance with ISO 11133:2014 requirements [20]: for BCYEα agar, colony count of positive strains was ≥70% for each inoculum (i.e., productivity); for MWY agar, colony count of positive strains was ≥50% for each inoculum (i.e., selectivity).

### 4.3. Laboratory Procedure

Briefly, the water samples were concentrated 100-fold by filtration through a 0.2-μm polycarbonate filter (Millipore, Billerica, MA, USA). The filter membrane was aseptically placed in one of the bottom corners inside the stomacher bag and rubbed with the finger and thumb of one hand for 1 min with 10 mL Page solution (pH 6.8) to detach the bacteria. A 0.2-mL volume of the concentrated sample was spread on duplicate plates of MWY or BCYEα agar. The plates were incubated at 36 °C in a humid 2.5% CO_2_ chamber and examined after 3, 6, and 10 days of incubation. Suspected colonies were subcultured on blood and BCYEα agar.

### 4.4. Identification of Legionella spp.

The presence of background flora was measured through semiquantitative counting. According to the visual density of the colonies spread onto the plate, four categories were determined, where zero represented no background flora and 3+ massive contamination (see supplementary materials). Colonies grown on MWY or BCYEα agar were subsequently identified by agglutination test (*Legionella* latex test; Oxoid). This test allows the separate identification of *L. pneumophila* Serogroup 1 and Serogroups 2 to 14 and detection of seven other species of *Legionella* (polyvalent). Colonies identified as *L. pneumophila* Serogroup 2 to 14 were further tested with *Legionella* agglutination latex reagents (Pro-Lab Diagnostics, Richmond Hill, Canada), which are intended for the identification of a single *L. pneumophila* sero group. Colonies not identified by the agglutination test were tested by polymerase chain reaction (in-house PCR) for the detection of the genus *Legionella*. This PCR assay utilizes specific primers to amplify the 16S rRNA gene of *Legionella* spp. [21]. The plate showing the highest number of confirmed colonies was used to estimate the number of *Legionella* spp. in the original sample (report). Concentrations of *Legionella* spp. in water samples are expressed as colony forming units per liter (CFU/l). According to this method, the lower limit of detection (LOD) is 50 CFU/L. 

### 4.5. Statistical Analysis

Agreement between the final reports (i.e., Oxoid and Xebios) and the two media (i.e., MWY and BCYEα) was assessed by two-by-two contingency tables, through Cohen’s κ coefficient. Comparison between *Legionella* spp. counts obtained by Xebios and Oxoid media was performed by a Wilcoxon signed-rank test.

To quantify the difference between counts on different media, our analyses took into account the decimal logarithm of counts observed. The median difference was chosen as the estimator of the actual difference for each comparison, as indicated by Wilcoxon’s nonparametric analysis.

The two 2 × 2 tables were compared using the Deming–Stephan method [22,23]. Specifically, the first table (Oxoid counts, see Table 3a) was transformed through the algorithm developed by Mosteller et al. [24] in order to obtain another table (Table 3c). These could be considered as the expected values for the Xebios table according to the results of the Oxoid count. Therefore, values in Table 4c were compared to the actual observed Xebios counts (Table 3b), with Pearson’s chi-squared test. 

The sensitivity of *Legionella* spp. detection was calculated by comparing the number of positive plates for a given medium with the cumulative yield of *Legionella* spp. from all four media. Selectivity for each method was defined as the number of plates that suppressed the growth of organisms that were not *Legionella* spp. Sensitivity and selectivity were compared using the exact McNemar test.

All statistical analyses were performed using the statistical software R (“stats” package, version 3.6.3) [25]. For all analyses, the level of significance was set at α = 0.05.

## 5. Conclusions

In conclusion, our data demonstrate that the quality of culture media is crucial in determining the level of *Legionella* spp. colonization in hospital water systems. As water remediation measures are based on quantitative *Legionella* spp. data obtained by culturing environmental samples, culture protocol standardization, as well as accurate quality control of the culture media, is essential to achieve intra- and interlaboratory reproducibility and accuracy.

Given the public health risk from *Legionella* spp., it is important that all water-testing laboratories carefully consider the following aspects: (1) there can be variability in *Legionella* spp. detection due to different types and brands of medium—of note, this variation can also be observed among different batches from the same supplier; (2) the medium should be purchased from a reputable company and fully validated in-house, with the inclusion of appropriate controls; (3) when switching to a different medium manufacturer, extensive validation should be performed in order to determine whether the new medium is “fit for purpose”.

## Figures and Tables

**Table 1:** Relative sensitivity and selectivity of BCYEα and MWY media from Xebios or Oxoid for *Legionella* spp. isolation.

	BCYEα ^Oxoid^	MWY ^Oxoid^	BCYEα ^Xebios^	MWY ^Xebios^
**Number (%) plates growing *Legionella***	44 (29.7)	52 (35.1)	49 (33.1)	62 (41.9)
**Sensitivity ^a^**	(63)	(74)	(70)	(78)
**Number (%) plates growing microorganism other than *Legionella***	21 (14.2)	70 (47.3)	18 (12.2)	70 (47.3)
**Selectivity ^b^**	(14)	(47)	(12)	(47)

**^a^** Sensitivity was calculated by comparing the number of positive plates for a given medium with the cumulative yield of *Legionella* spp. from all four media (n = 70). **^b^** Selectivity for each media was defined as the number of plates suppressing the growth of organisms that were not *Legionella* spp. over the total number of plates (n = 148).

**Table 2:** Wilcoxon signed-rank test analysis of *Legionella* spp. counts (CFU/L) obtained with four different types of mediums.

	Xebios Media vs. Oxoid Media		*P*-Value	Δ (CI_95%_)
**(a)**	BCYEα ^Oxoid^	MWY ^Oxoid^	0.476	
**(b)**	BCYEα ^Xebios^	MWY ^Xebios^	0.0005	Δ = 0.515 (CI_95%_: 0.182, 1.023) [MWY > BCYE]
**(c)**	BCYEα ^Oxoid^	BCYEα ^Xebios^	0.0905	
**(d)**	MWY ^Oxoid^	MWY ^Xebios^	0.0014	Δ = 0.618 (CI_95%_: 0.268, 0.981) [Xebios > Oxoid]

Comparison between corresponding media from the two companies (a,b) and between different types of medium from each company (c,d). Δ is the median of the differences of log-counts, where the left column is the reference.

**Table 3:** Assessment of *Legionella* spp. recovery according to culture medium and manufacturer.

	(a) OXOID				(b) XEBIOS				(c) EXPECTED XEBIOS [from (a) with Deming-Stephan Method]			
	BCYEα				BCYEα				BCYEα			
**MWY**		**Pos** **(n)**	**Neg** **(n)**	**Total**		**Pos** **(n)**	**Neg** **(n)**	**Total**		**Pos** **(n)**	**Neg** **(n)**	**Total**
	**Pos** **(n)**	34	18	52	**Pos** **(n)**	47	15	62	**Pos** **(n)**	40.22	21.78	62
	**Neg** **(n)**	10	86	96	**Neg** **(n)**	2	84	86	**Neg** **(n)**	8.78	77.22	86
	**Total**	44	104	148	**Total**	49	99	148	**Total**	49	99	148

Pos = positive, Neg = negative Agreement = 81.1%; κ = 0.57 Agreement = 88.5%; κ = 0.757.

**Table 4:** Number of *Legionella* spp. colonies (CFU/L) appearing on different plates: BCYEα positive results vs. MWY negative results.

Oxoid						Xebios					
Samples	BCYEα	MWY	*L*. spp.	B. Flora on BCYEα	B. Flora on MWY	Samples	BCYEα	MWY	*L.* spp.	B. Flora on BCYEα	B. Flora on MWY
**1**	50	0	Lp3	1+	0	**1**	50	0	Lp3	0	/
**2**	50	0	Lp1	1+	0	**2**	500	0	Lp1	1+	/
**3**	150	0	Lp6	1+	0						
**4**	200	0	L.spp	0	0						
**5**	400	0	L.spp	0	0						
**6**	600	0	L.spp	1+	0						
**7**	1050	0	L.spp	0	0						
**8**	2200	0	L.spp	2+	0						
**9**	2350	0	L.spp	0	0						
**10**	3400	0	L.spp	0	0						

Background (B.) flora was measured by semiquantitative counting: four categories were determined according to the visual density of colonies spread onto the plate, where zero is no background flora and 3+ is massive contamination (see supplementary materials). Lp 1 = *L. pneumophila* serogroups 1; Lp 3 = *L. pneumophila* serogroups 3; Lp 6 = *L. pneumophila* serogroups 6; L.spp. = *Legionella* spp. non-*pneumophila.*

**Table 5:** Number of *Legionella* spp. colonies (CFU/L) appearing on different plates: BCYEα negative results vs. MWY positive results.

Oxoid						Xebios					
Samples	BCYEα	MWY	*L.* spp.	B. Flora on BCYEα	B. Flora on MWY	Samples	BCYEα	MWY	*L.* spp.	B. Flora on BCYEα	B. Flora on MWY
**1**	0	50	Lp6	3+	0	**1**	0	50	Lp6	3+	0
**2**	0	50	L.spp	2+	0	**2**	0	50	Lp3, L.spp	1+	1+
**3**	0	50	Lp6	1+	0	**3**	0	50	L.spp	1+	0
**4**	0	50	Lp6	3+	0	**4**	0	50	Lp6	3+	0
**5**	0	50	Lp6	3+	0	**5**	0	100	Lp6	2+	0
**6**	0	100	Lp1	1+	0	**6**	0	500	Lp7-14	3+	0
**7**	0	150	Lp6	2+	0	**7**	0	500	Lp7-14	3+	0
**8**	0	200	Lp7-14	3+	0	**8**	0	800	Lp6, L.spp	2+	0
**9**	0	300	Lp7-14	3+	0	**9**	0	1200	Lp7-14	3+	1+
**10**	0	300	Lp714	3+	0	**10**	0	1300	Lp714	3+	1+
**11**	0	300	Lp7-14	3+	0	**11**	0	1500	Lp7-14	3+	1+
**12**	0	300	Lp6	3+	0	**12**	0	1700	Lp6, L.spp	3+	0
**13**	0	300	Lp6	3+	0	**13**	0	2150	Lp3, Lp6	3+	1+
**14**	0	500	Lp7-14	3+	0	**14**	0	7700	Lp6	3+	1+
**15**	0	600	Lp7-14	3+	0	**15**	0	8300	Lp6	3+	0
**16**	0	1500	Lp3, Lp6	3+	0						
**17**	0	4800	Lp2-14	3+	0						
**18**	0	15000	Lp6	3+	3+						

Background (B.) flora was measured by semiquantitative counting: four categories were determined according to the visual density of colonies spread onto the plate, where zero is no background flora and 3+ is massive contamination (see supplementary materials). Lp 1 = *L. pneumophila* serogroups 1; Lp 3 = *L. pneumophila* serogroups 3; Lp 6 = *L. pneumophila* serogroups 6; Lp 2–14 = *L. pneumophila* serogroups 2–14; Lp 7–14= *L. pneumophila* serogroups 7–14; L.spp = *Legionella* spp. non-*pneumophila*.

## Supplementary Materials

The following are available online at https://www.mdpi.com/2076-0817/9/7/523/s1. File: images of plate with background flora; paired images (BCYEα and MWY agar by two manufacturers) obtained during incubation period of inoculated plates.

## References

[1] International Organization for Standardization (1998). ISO 11731: 1998 Water Quality—Detection and Enumeration of Legionella.

[2] Centers for Disease Control and Prevention (CDC) (2005). Legionnaires Disease Procedures Manual for Recovery | Legionnaires.

[3] Health Protection Agency (HPA) (2006). Detection and Enumeration of Legionella Species by Filtration and Centrifugation.

[4] Lück P.C., Igel L., Helbig J.H., Kuhlisch E., Jatzwauk L. (2004). Comparison of commercially available media for the recovery of Legionella species. Int. J. Hygiene Environ. Health.

[5] Edelstein P.H., Edelstein M.A.C. (2010). Comparison of the plating efficiencies and shelf lives of three different commercial buffered charcoal yeast extract media supplemented with alpha-ketoglutaric acid. J. Clin. Microbiol..

[6] Mossel D.A., Bonants-Van Laarhoven T.M., Ligtenberg-Merkus A.M., Werdler M.E. (1983). Quality assurance of selective culture media for bacteria, moulds and yeasts: An attempt at standardization at the international level. J. Appl. Bacteriol..

[7] Feeley J.C., Gibson R.J., Gorman G.W., Langford N.C., Rasheed J.K., Mackel D.C., Baine W.B. (1979). Charcoal-yeast extract agar: Primary isolation medium for Legionella pneumophila. J. Clin. Microbiol..

[8] Edelstein P.H. (1981). Improved semiselective medium for isolation of Legionella pneumophila from contaminated clinical and environmental specimens. J. Clin. Microbiol..

[9] Ditommaso S., Gentile M., Giacomuzzi M., Zotti C.M. (2011). Recovery of Legionella species from water samples using an internal method based on ISO 11731: Suggestions for revision and implementation. Diagn. Microbiol. Infect. Dis..

[10] International Organization for Standardization (2017). ISO 11731: 2017 Water Quality—Enumeration of Legionella.

[11] Boulanger C.A., Edelstein P.H. (1995). Precision and accuracy of recovery of Legionella pneumophila from seeded tap water by filtration and centrifugation. Appl. Environ. Microbiol..

[12] Ta A.C., Stout J.E., Yu V.L., Wagener M.M. (1995). Comparison of culture methods for monitoring Legionella species in hospital potable water systems and recommendations for standardization of such methods. J. Clin. Microbiol..

[13] Brenner K.P., Rankin C.C. (1990). New screening test to determine the acceptability of 0.45-micron membrane filters for analysis of water. Appl. Environ. Microbiol..

[14] Smith L., Carroll K., Mottice S. (1993). Comparison of membrane filters for recovery of legionellae from water samples. Appl. Environ. Microbiol..

[15] Mossel D.A. (1991). Food microbiology: An authentic academic discipline with substantial potential benefits for science and society. J. Assoc. Anal. Chem..

[16] Wadowsky R.M., Yee R.B. (1981). Glycine-containing selective medium for isolation of Legionellaceae from environmental specimens. Appl. Environ. Microbiol..

[17] Edelstein P.H. (1982). Comparative study of selective media for isolation of Legionella pneumophila from potable water. J. Clin. Microbiol..

[18] Hammer B.K., Swanson M.S. (1999). Co-ordination of legionella pneumophila virulence with entry into stationary phase by ppGpp. Mol. Microbiol..

[19] Miyamoto H., Yamamoto H., Arima K., Fujii J., Maruta K., Izu K., Shiomori T., Yoshida S. (1997). Development of a new seminested PCR method for detection of Legionella species and its application to surveillance of legionellae in hospital cooling tower water. Appl. Environ. Microbiol..

[20] International Organization for Standardization (2014). ISO 11133: 2014 Microbiology of Food, Animal Feed and Water—Preparation, Production, Storage and Performance Testing of Culture Media.

[21] Edelstein P.H., Edelstein M.A. (1991). Comparison of different agars used in the formulation of buffered charcoal yeast extract medium. J. Clin. Microbiol..

[22] Deming W.E., Stephan F.F. (1940). On a Least Squares Adjustment of a Sampled Frequency Table When the Expected Marginal Totals are Known. Ann. Math. Stat..

[23] Altham P.M.E., Ferrie J.P. (2007). Comparing Contingency Tables Tools for Analyzing Data from Two Groups Cross-Classified by Two Characteristics. Hist. Methods J. Quant. Interdiscip. Hist..

[24] Mosteller F. (1968). Association and Estimation in Contingency Tables. J. Am. Stat. Assoc..

[25] R Development Core Team (2019). A Language and Environment for Statistical Computing.

