# Supplementary material for: Sensitivity and Selectivity of Two Commercially Available Media for Legionella spp. Recovery from Environmental Water Samples
Source: Pathogens. 2020 Jun 29;9(7):523. doi: 10.3390/pathogens9070523 (PMC7400336; doi:10.3390/pathogens9070523)

Examples of plates with different level of background flora: from complete absence (zero) to massive contamination (3+)

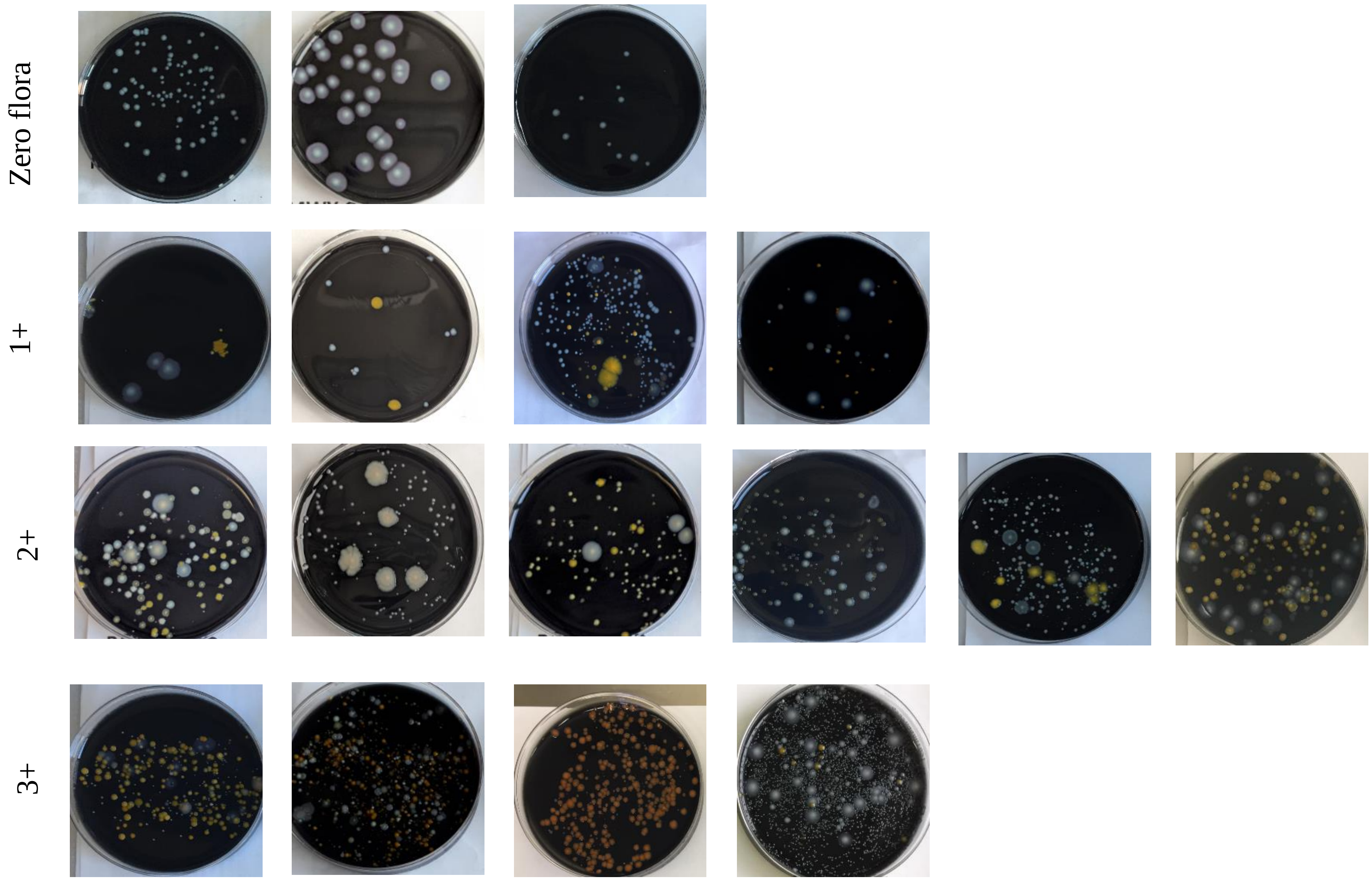

Each pair of plates represents <u>concordant positive samples</u> cultured on **MWY** media of the two different companies.
(the plate at the top is the Xebios one , Oxoid’s is the one at the bottom).

In these plates colony size were greater for bacteria plated onto Xebios media, whilst colony count was almost the same.

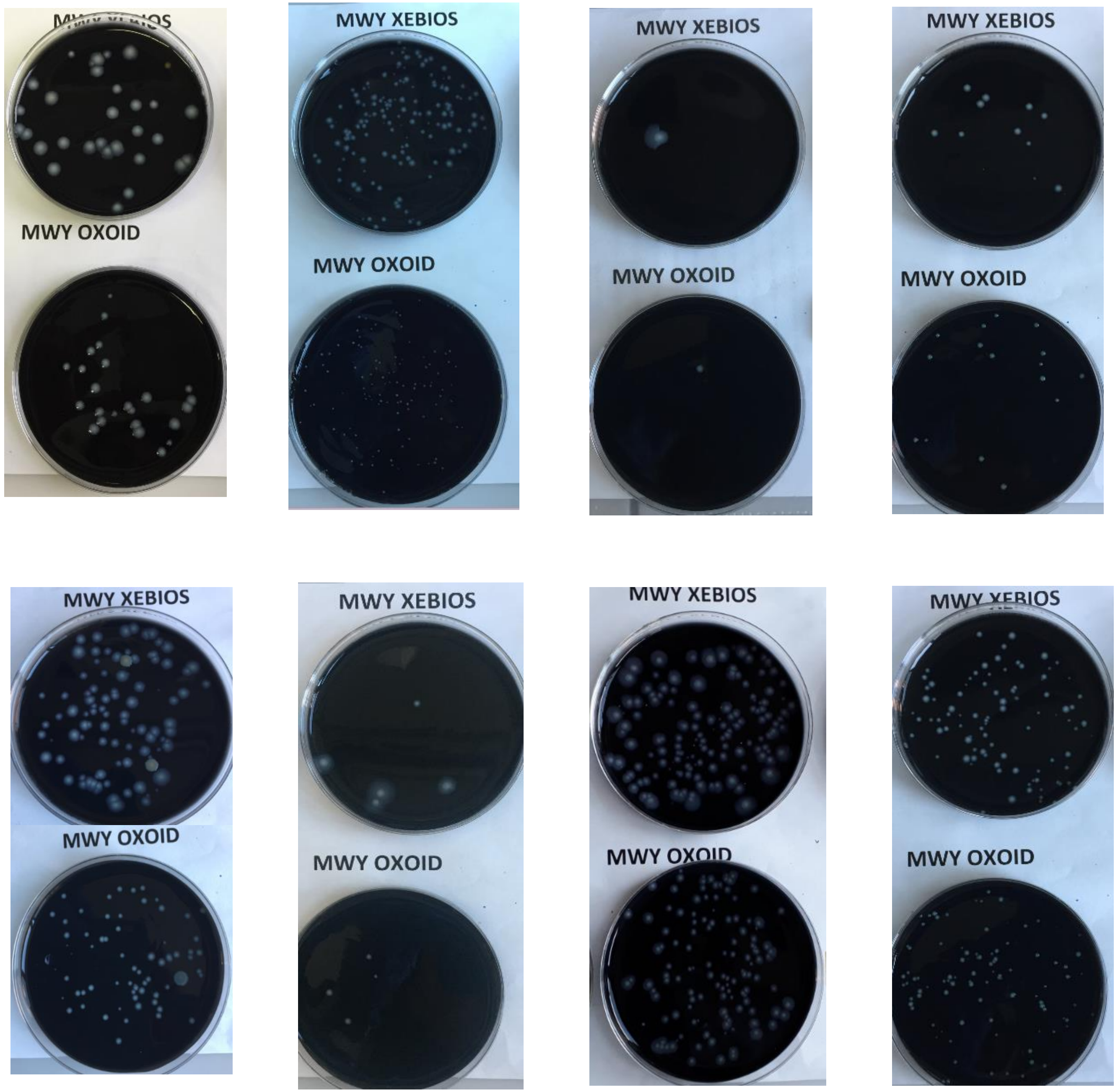

Each pair of plates represents concordant positive samples cultured on **MWY** media of the two different companies.
(the plate at the top is the Xebios one , Oxoid's is the one at the bottom).

In these plates both colony size and colony count were greater for bacteria plated onto Xebios media.

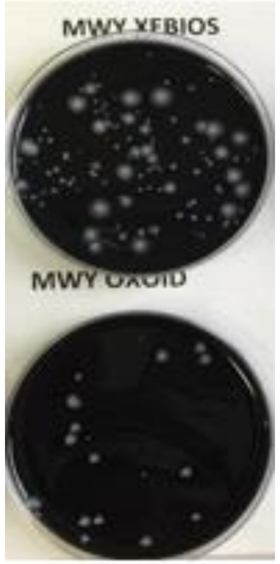

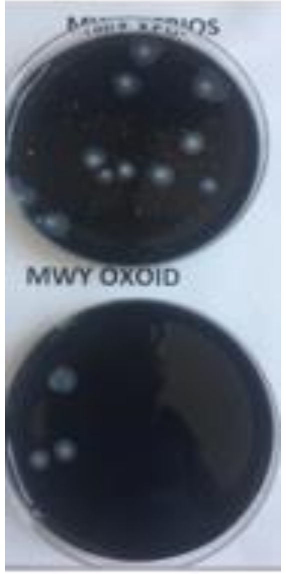

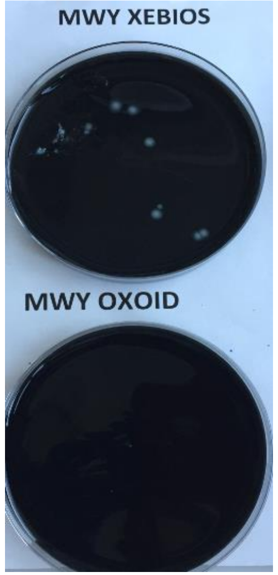

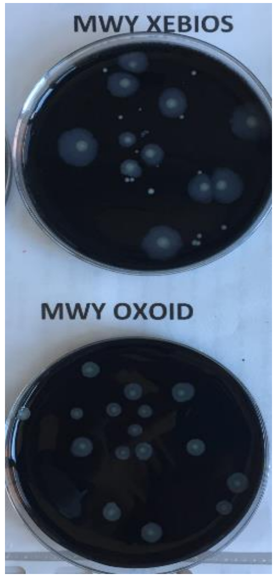

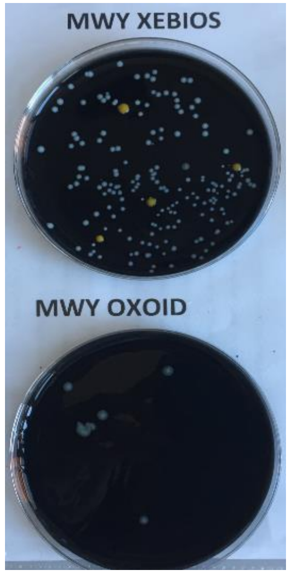

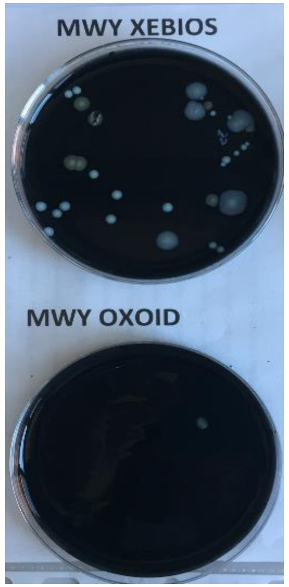

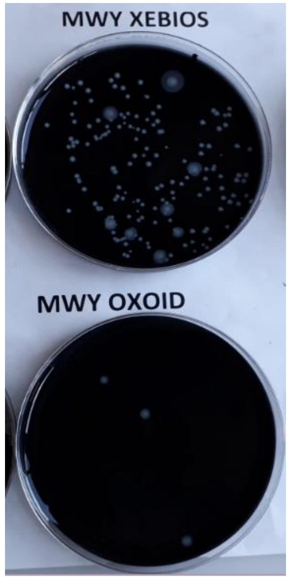

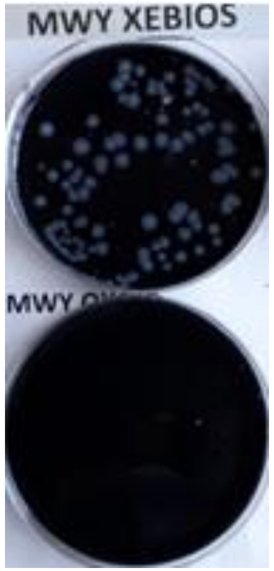

Each pair of plates represents concordant positive samples cultured on **BCYEα** media of the two different companies.
(the plate at the top is the Xebios one , Oxoid's is the one at the bottom).

In these plates both colony size and colony count were almost the same on **BCYEα** media of the two companies.

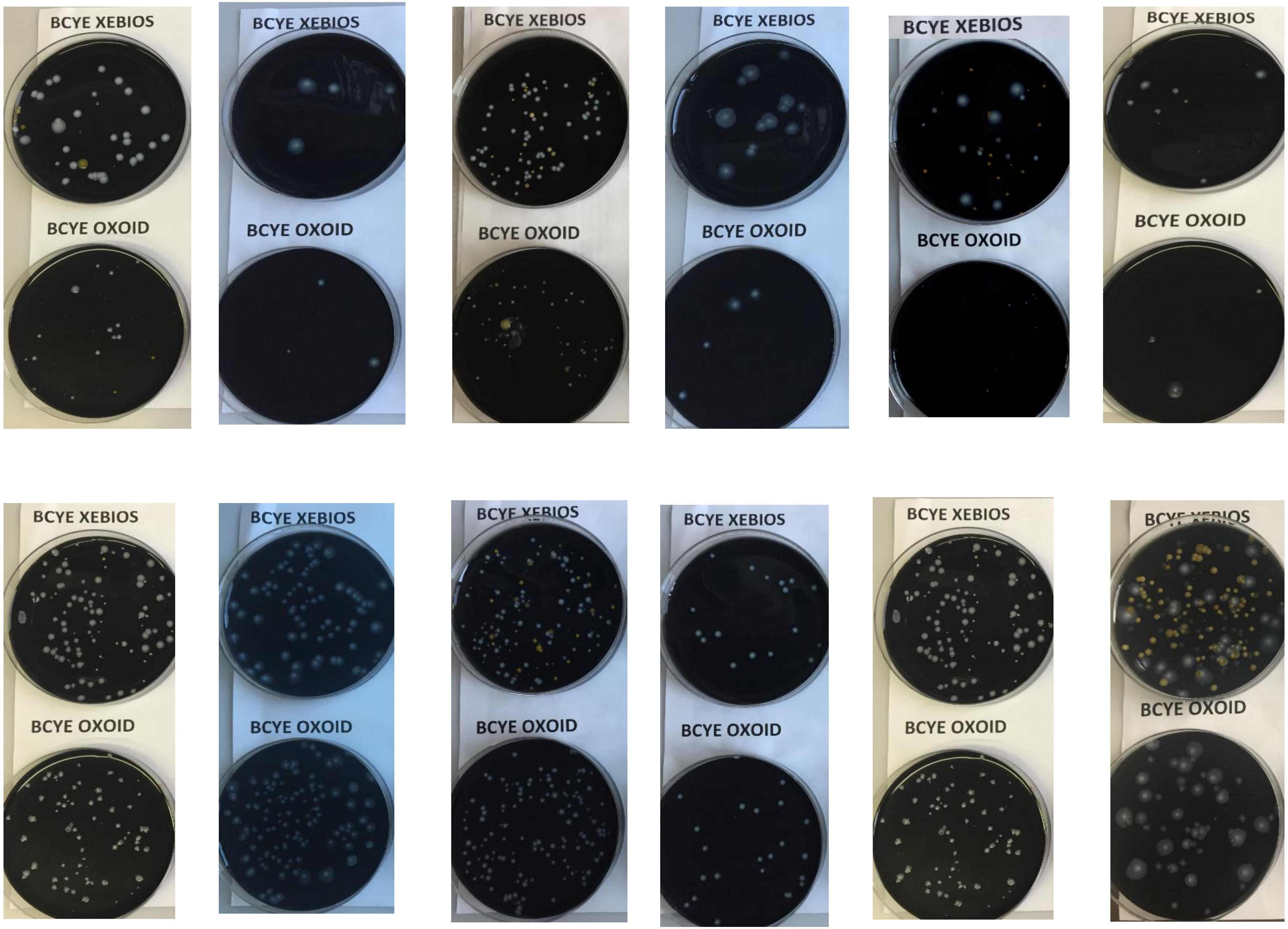

Selective **MWY** agar media affected the recovery of *Legionella* species non-*pneumophila*, only in samples cultured on Oxoid media.
(the plate at the top is the Xebios one , Oxoid’s is the one at the bottom).

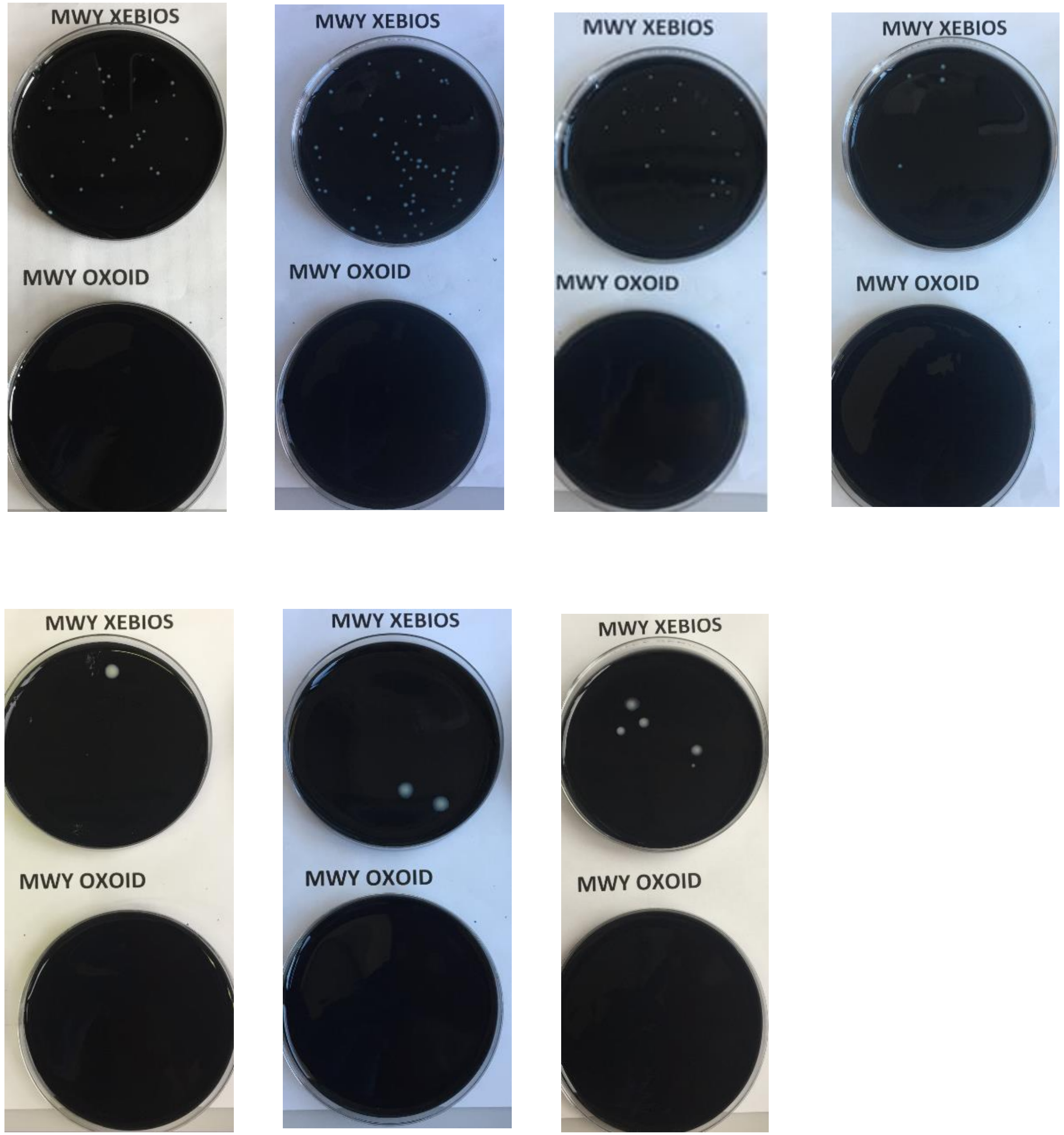

Supplement: Supplementary file 1 [file pathogens-09-00523-s001.pdf]
